# Similar quality in chronic kidney disease multidisciplinary follow-up between kidney

**DOI:** 10.1590/2175-8239-JBN-2019-0239

**Published:** 2020-12-18

**Authors:** Moisés Carminatti, Natália Maria Silva Fernandes, Fernando Antonio Basile Colugnati, Helady Sanders-Pinheiro

**Affiliations:** 1 Universidade Federal de Juiz de Fora, Hospital Universitário, Divisão de Nefrologia, unidade de Transplante Renal, Juiz de Fora, MG, Brasil.; 2 Núcleo Interdisciplinar de Estudos e Pesquisas em Nefrologia, Juiz de Fora, MG, Brasil.

**Keywords:** Renal Insufficiency, Chronic, Kidney Transplantation, Patient Care Team, Health Services Research, Graft Survival., Insuficiência Renal, Crônica, Transplante Renal, Equipe de Assistência ao Paciente, Pesquisa de Serviços de Saúde, Sobrevivência de Enxerto.

## Abstract

**Introduction:**

Multidisciplinary clinics are the best approach towards Chronic Kidney Disease (CKD) patients in pre-dialysis phases. The few studies regarding kidney transplant recipients (KTR) compare multidisciplinary and non-multidisciplinary clinics.

**Methods:**

In this study, we compared the quality of multidisciplinary CKD care between 101 KTR and 101 propensity score-matched non-transplant pre-dialysis patients (PDP). Prevalence of patients without specific treatment at any time and percent time without specific treatment for CKD complications were the main outcomes and patient and kidney function survival, glomerular filtration rate (GFR) decline, prevalence of CKD-related complications, and percent time within therapeutic goals were the exploratory ones.

**Results:**

Time within most goals was similar between the groups, except for diastolic blood pressure (83.4 *vs*. 77.3%, RR 0.92, CI 0.88-0.97, *p* = 0.002) and hypertriglyceridemia (67.7 *vs*. 58.2%, OR 0.85, CI 0.78-0.93, *p* < 0.001), better in non-transplant PDP, and for proteinuria (92.7 *vs*. 83.5%, RR 1.1, CI 1.05-1.16, *p* < 0.001), better in KTR. Patient survival and GFR decline were similar between the groups, although non-transplant PDP tended to progress earlier to dialysis (9.9% *vs*. 6.9%, HR 0.39, *p* = 0.07, CI 0.14-1.08).

**Discussion:**

The similar findings between non-transplant PDP and KTR suggests that good and comparable quality of multidisciplinary is a valid strategy for promoting optimal clinical management of CKD-related complications in KTR.

## Resumo

**Introdução:**

Clínicas multidisciplinares são a melhor abordagem para pacientes com doença renal crônica (DRC) em fases pré-dialíticas. Os poucos estudos sobre receptores de transplante renal (RTR) comparam clínicas multidisciplinares e não multidisciplinares.

**Métodos:**

Neste estudo, comparamos a qualidade do atendimento multidisciplinar para DRC entre 101 RTR e 101 pacientes pré-dialíticos (PPD) não transplantados pareados com escore de propensão. A prevalência de pacientes sem tratamento específico em qualquer momento e a porcentagem de tempo sem tratamento específico para complicações de DRC foram nossos desfechos principais, e a sobrevida do paciente e da função renal, declínio da taxa de filtração glomerular (TFG), prevalência de complicações relacionadas à DRC e porcentagem de tempo dentro dos objetivos terapêuticos foram os exploratórios.

**Resultados:**

O tempo no alvo para a maioria dos objetivos foi semelhante entre os grupos, exceto para a pressão arterial diastólica (83,4 *vs*. 77,3%, RR 0,92, IC 0,88-0,97, *p* = 0,002) e hipertrigliceridemia (67. 7 *vs*. 58,2%, OR 0,85, IC 0,78-0,93, *p* < 0,001), melhor em PPD não transplantados, e para proteinúria (92,7 *vs*. 83,5%, RR 1,1, IC 1,05-1,16, *p* < 0,001), melhor em RTR. A sobrevida do paciente e o declínio da TFG foram semelhantes entre os grupos, embora PPD não transplantados tendessem a progredir mais cedo para a diálise (9,9% vs. 6,9%, HR 0,39, *p* = 0,07, IC 0,14-1,08).

**Discussão:**

Os resultados semelhantes entre PPD não transplantados e os RTR sugerem que a qualidade multidisciplinar boa e comparável é uma estratégia válida para promover a gestão clínica ideal de complicações relacionadas à DRC em RTR.

## Introduction

Kidney transplant is the best modality of renal replacement therapy (RRT) for patients with end-stage chronic kidney disease (CKD), providing lower mortality rate, better quality of life, and better control of CKD-related complications and comorbidities, such as hypertension, anemia, bone mineral disorder, metabolic acidosis, and hypervolemia.^1^^,^ 2

As stated in the Kidney Disease Improving Global Outcomes (KDIGO) guidelines, kidney transplant recipients (KTR) are a particular subset of patients with CKD. These patients, in addition to alloimmune phenomena and potentially life-threatening side effects from immunosuppressive drugs, may also undergo CKD progression and dialysis. During this process, KTR experience severe endothelial derangement, with enhanced risk of hard cardiovascular endpoints and progression to category 5 CKD, similar to non-transplant pre-dialysis patients (NT-PDP).^3^^-^ 5

Long-term patient and graft survival have modestly improved over recent decades.^6^^,^ 7 Alloimmune risks, including human leukocyte antigen (HLA) incompatibility, exposure to donor-specific antibodies, rejection episodes, and graft function at 1 year post-transplant are the major determinants of long-term kidney function survival. However, there is growing interest in classical clinical factors such as hypertension, proteinuria, anemia, diabetes, dyslipidemia, bone mineral disorders, and metabolic acidosis that contribute to CKD progression, notably after the first year post-transplant.^8^^-^ 10

Multidisciplinary clinics are the best model for clinical management of NT-PDP, but insufficient attention is directed at classical CKD-related complications in KTR, which are all classified as pre-dialysis patients (PDP).^5^ Few studies describe the impact of multidisciplinary approach on the treatment of CKD in KTR, mostly through comparisons between multidisciplinary and non-multidisciplinary clinics.^11^^-^ 14 The present study compares the quality of treatment of CKD-related complications between KTR and NT-PDP and explores the CKD progression when both cohorts are followed in multidisciplinary clinics.

## Methods

**Figure 1:**
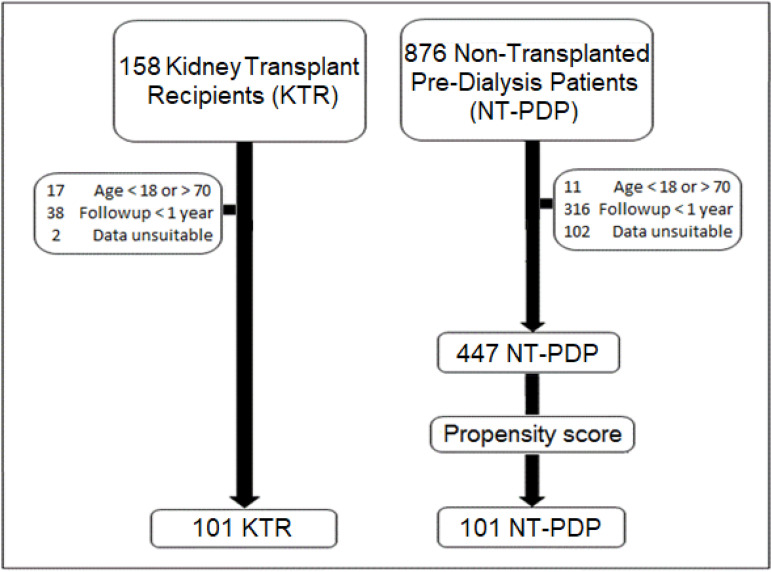
Sample composition. Patient selection according to inclusion and exclusion criteria.

This retrospective study included patients followed at the Nephrology unit of the Federal University of Juiz de Fora, Brazil, between January 1, 2010 and December 31, 2014. At this CKD clinic, a multidisciplinary team of nephrologists, nurses, dietitians, social assistants, and psychologists routinely assist all NT-PDP and KTR. At each office visit, the whole multidisciplinary team evaluated all scheduled patients following a detailed care program, defined based on published guidelines, adapted after discussion with local facilities' administrators.^5^^,^ 15 Visit intervals were individualized and planed to be no longer than three months. Inclusion criteria were: PDP in categories 1 to 5, age 18-70 years (upper limit to KT), follow-up > 1 year post-transplant for KTR, and > 1 year of clinic follow-up for NT-PDP. Exclusion criteria were lack of birthdate, weight, height, transplant date for KTR, or at least two measures of serum creatinine plus two systolic (SBP) and diastolic blood pressure (DBP) measurements in the first follow-up year. Of 876 NT-PDP and 158 KTR, 447 NT-PDP and 101 KTR matched inclusion criteria and were selected for the study. Furthermore, 101 of 447 NT-PDP were selected through a "nearest neighbor" propensity score-matching (PSM) model (considering age, sex, race, body mass index (BMI), obesity, CKD category, hypertension, diabetes, coronary artery disease, cerebrovascular disease, peripheral artery disease, and congestive heart failure), resulting in a study sample of 101 NT-PDP and 101 KTR (Figure 1).^16^

Demographic data included age, sex, race, etiology of CKD, comorbidities (hypertension, diabetes, obesity, cardiovascular disease, and smoking), and specific characteristics of KTR (dialysis duration, donor type, HLA matches, and immunosuppressive drugs). Estimated glomerular filtration rate (eGFR) was calculated with the Chronic Kidney Disease Epidemiology Collaboration (CKD-EPI) formula.^17^

CKD-related complications were defined as: systolic hypertension (> 140 mmHg, or > 130 mmHg in diabetic patients with proteinuria > 300 mg/24h, or use of anti-hypertensives), diastolic hypertension (> 90 mmHg, or > 80 mmHg in diabetic patients with proteinuria > 300 mg/24h, or use of anti-hypertensives), clinically significant proteinuria (> 1 g/24h), anemia (hemoglobin <11 g/dL until December 31^st^, 2013, or < 10 g/dL after January 1^st^, 2014, or use of erythropoietin), hypocalcaemia (calcium < 8.5 mg/dL), hyperphosphatemia (phosphate > 4.5 mg/dL for patients in CKD categories 1-4, or > 5.5 mg/dL for patients in CKD category 5, or phosphate chelation), hyperparathyroidism (PTH > 450 pg/mL, or use of 1,25-OH-vitamin D), hypovitaminosis D (25-OH-vitamin D < 30 ng/mL, or use of 25-OH-vitamin D), hypercholesterolemia (total cholesterol > 200 mg/dL, or use of statins), elevated LDL (> 100 mg/dL, or use of statins), low HDL (< 50 mg/dL for women and < 55 mg/dL for men), hypertriglyceridemia (> 150 mg/dL), hyperuricemia (> 8.0 mg/dL, or use of allopurinol), and metabolic acidosis (bicarbonate <22 mEq/L, or use of sodium bicarbonate), according to the Brazilian and international guidelines for CKD management.^5^^,^ 15

We compared the percentage of KTR and NT-PDP patients with CKD-related complications, both at baseline and throughout follow-up. Next, we assessed the percentage of follow-up visits wherein KTR and NT-PDP patients received specific treatments for those complications to measure treatment quality. We compared the percentage of follow-up visits wherein KTR and NT-PDP patients were within therapeutic goals to measure treatment performance.

Statistical analysis consisted of a comparative description of clinical and laboratory characteristics between the cohorts, by means (± standard deviation) or medians (and range), after analyzing sample normality using the Kolmogorov-Smirnov and Shapiro-Wilk tests, and determining categorical variable frequencies. As the main outcome for evaluating the CKD care quality between the two groups, we considered the percentage of follow-up visits wherein KTR and NT-PDP patients received specific treatments. In addition, as exploratory outcomes of treatment performance, we accounted for the percentage of follow-up visits wherein KTR and NT-PDP patients were within therapeutic goals and patient and dialysis-free kidney function survival. For each clinical complication and untreated complication, we assessed frequencies, odds ratios, or relative risks, 95% confidence intervals (CI) and p values. Chi-square or t-tests were used for each subset of variables. Kaplan-Meier analysis was employed to assess patient and dialysis-free kidney function survival compared by log-rank testing. A mixed linear model permitted comparative analysis of GFR decay between the two cohorts. SPSS 20.0 (IBM, Chicago, Illinois, USA), Stata 13.0 (Stata Corporation, College Station, Texas, USA) and MedCalc (MedCalc Software, B-8400, Ostend, Belgium) were used for the analyses.

The study was approved by the local Ethics Committee (approval number 275/2011, December 15^th^, 2011), and was conducted in accordance with ethical standards of the 1975 Helsinki Declaration and later amendments or comparable standards. Informed consent was waived by the local ethics committee.

## Results

The study included 101 NT-PDP and 101 KTR (Figure 1). After PSM selection of NT-PDP, baseline characteristics such as GFR, cardiovascular comorbidities, and CKD category distribution were matched. KTR were younger than NT-PDP (43.4±12.5 *vs*. 50.2±13.5 years), with lower BMI (24.7±4.4 *vs*. 26.1±4.4), and longer follow-up (55.7±12.1 *vs*. 31.6±11.5 months). Among NT-PDP, the predominant cause of CKD was hypertensive nephrosclerosis, whereas in KTR it was chronic glomerulonephritis (Table 1).

**Table 1:** Demographic and clinical characteristics of NT-PDP and KTR

	NT-PDP (N = 101)	KTR (N = 101)	P
**Demographics**			
Age (years)	50.2 ± 13.5	43.4 ± 12.5	<0.001
Female sex (%)	37.6	31.7	0.46
Caucasian race (%)	54.4	74.2	0.003
Body mass index (kg/m^2^)	26.18	24.78	0.025
Follow-up (months)	31.61	55.77	<0.001
**Renal function**			
Creatinine (mg/dL)	1.62 ± 0.61	1.59 ± 0.53	0.735
eGFR (mL/min/1.73m^2^)	51.69 ± 20.18	53.27 ± 16.88	0.548
**CKD category at baseline (%)**			0.509
1	2.9	2.9	
2	24.8	29.7	
3a	40.6	36.6	
3b	22.8	24.8	
4	8.9	5.9	
5	0	0	
**Primary cause of CKD (%)**			0.397
Hypertensive	43.6	20.8	
Glomerulonephritis	12.9	40.6	
Diabetes	4.0	2.0	
Adult polycystic kidney disease	11.9	5.0	
Other	6.9	5.9	
Undetermined	20.8	25.7	
**Comorbidities at Baseline (%)**			
Hypertension	92.1	86.1	0.175
Diabetes	4.95	2.97	0.251
Obesity	20.8	11.9	0.088
Coronary artery disease	6.93	6.93	1.0
Peripheral artery disease	2.97	0.99	0.315
Cerebrovascular disease	4.95	1.98	0.251
Congestive heart failure	5.94	3.96	0.519
Smoking	9.9	8.9	0.808
**Baseline clinical characteristics**			
Systolic blood pressure (mmHg)	137.6 ± 25.3	125.1 ± 13.1	<0,001
Diastolic blood pressure (mmHg)	85.5 ± 13.9	80.0 ± 11.0	0.002
Haemoglobin (g/dL)	13.5 ± 1.5	12.7 ± 1.67	< 0.001
Proteinuria (mg/24h)	169(10-5650)	184(15-1500)	0.071
Total cholesterol (mg/dL)	189.8 ± 43.4	186.6 ± 37.8	0.598
LDL cholesterol (mg/dL)	115.0 ± 35.8	109.7 ± 28.3	0.291
HDL cholesterol (mg/dL)	50.4 ± 14.7	46.4 ± 13.5	0.056
Triglycerides (mg/dL)	138.1 ± 78.1	151.4 ± 96.8	0.33
Calcium (mg/dL)	9.4 ± 0.7	9.7 ± 0.7	0.033
Phosphorus (mg/dL)	3.6 ± 0.7	3.4 ± 0.8	0.124
PTH (pg/mL)	63 (7.6-784)	76.7 (19-530)	0.886
Cholecalciferol (ng/mL)	23.5 (2-42)	24.4 (16.7-38)	0.872
Albumin (g/dL)	4.0 ± 0.5	4.2 ± 0.5	0.144
Bicarbonate (mEq/L)	24.6 ± 3.6	23.3 ± 2.6	0.113
Uric Acid (mg/dL)	6.6 ± 1.6	6.3 ± 1.5	0.246
**Overall use of key medications (%)**			
Anti-hypertensives	94.0	90.1	0.298
ACEi or ARB	87.1	78.2	0.127
Betablockers	45.5	38.6	0.32
ASA	17.8	15.8	0.707
Statins	49.5	61.3	0.092
Phosphate binder	5.94	4.95	0.757
1,25 OH Vitamin D	4.95	2.97	0.476
Cholecalciferol	48.5	2.97	< 0.001
Erythropoietin	3.96	28.7	< 0.001
Bicarbonate	14.8	19.8	0.355
Allopurinol	16.8	10.9	0.227

^1^Data are shown as percentages, means ± standard deviation, or medians. Continuous variables were compared using the t-test or Mann-Whitney U test, and frequencies were compared using the Chi-square test or Fisher test. 2 NT-PDP: non-transplanted pre-dialysis patients; KTR: kidney transplant recipients; eGFR: estimated glomerular filtration rate, according to the CKD-EPI formula; CKD: chronic kidney disease; ACEi: angiotensin-converting enzyme inhibitors; ARB: angiotensin receptor blocker; ASA: acetylsalicylic acid; LDL: low-density lipoprotein; HDL: high-density lipoprotein; PTH: parathyroid hormone.

Most KTR had living related-donors (84.2%), and 3 HLA matches (54.5%). The median time spent on dialysis was 20 months (0-112 months), and 12 transplants were pre-emptive. Acute rejection was observed in 9.9%, and new-onset diabetes in 17.8%. Immunosuppression more often comprised prednisone (89.1%), mycophenolate (73.2%), and tacrolimus (56.4%). Most KTR were on triple therapy (89.1%), and 79.1% received calcineurin inhibitors (Table 2). Prescription of medications for CKD-related complications was similar between groups, except for erythropoietin, more common in KTR (28.7% *vs.* 3.96%, *p* < 0.001), and cholecalciferol, more common in NT-PDP (48.5% *vs*. 2.97%, *p* < 0.001) (Table 1).

**Table 2:** Specific clinical characteristics of KTR

Donor Type (%)	
Living related	84.2
Living unrelated	11.9
Deceased	3.9
	
**HLA matches (%)**	
0-2	26.7
3	54.5
6	18.8
**Median time on dialysis (months)**	20 (0 – 112, 12 preemptive transplants)
**Complications during follow-up (%)**	
Delayed graft function	5.94
Acute rejection	9.9
Post-transplant Diabetes	17.8
**Immunosuppression (%)**	
Prednisone	89.1
Tacrolimus	56.4
Cyclosporine	23.7
Mycophenolate	73.2
Azathioprine	26.7
Rapamycin	32.6
Everolimus	8.9
Triple medication	89.1
Calcineurin inhibitor	79.1

^1^Data are shown as percentages or as means ± standard deviation. ^2^KTR: kidney transplant recipients; HLA: human leukocyte antigen.

At baseline, mean SBP (137.6±25.3 *vs*. 125.1±13.1 mmHg, *p* < 0.001) and DBP (85.5±13.9 *vs*. 80.0±11.0 mmHg, *p* = 0.002) were higher in NT-PDP (Table 1). There was a trend toward a higher prevalence of both systolic and diastolic hypertension in NT-PDP at baseline, but those differences were not observed throughout the follow-up (Table 3). Percent time within SBP therapeutic goal was similar between groups, whereas DBP was more often within goal in NT-PDP (83.4 vs. 77.3%, RR 0.92, CI 0.88-0.97, *p* = 0.002).

**Table 3:** CKD-related complications, treatment distribution, and achievement of specific therapeutic goals in NT-PDP and KTR groups throughout follow-up

	NT-PDP (%)	KTR (%)	RR/OR	CI	*p*
**Hypertension and blood pressure** **control**					
Baseline systolic hypertension	92.1	85.1	0.49*	0.19-1.22	0.126
Systolic hypertension in follow-up	94.1	92.1	0.97	0.9-1.05	0.579
Systolic BP within goal	75.7	76.5	1.01	0.95-1.06	0.695
Baseline diastolic hypertension	92.1	86.1	0.53*	0.21-1.33	0.18
Diastolic hypertension in follow-up	94.1	93.1	0.98	0.92-1.06	0.774
Diastolic BP within goal	83.4	77.3	0.92	0.88-0.97	0.002
**Proteinuria and anaemia**					
Baseline proteinuria > 1g/day	11.8	7.9	0.64*	0.24-1.67	0.36
Proteinuria > 1g/day in follow-up	22.6	23.7	1.05	0.62-1.75	0.845
Patients with untreated proteinuria	3.2	6.9	2.14	0.57-8.06	0.257
Time with untreated proteinuria	7.5	17.2	2.27	0.76-6.73	0.137
Proteinuria within goal	83.5	92.7	1.1	1.05-1.16	< 0.001
Baseline anaemia	6.9	22.8	3.95*	1.61-9.71	0.002
Anemia in follow-up	15.8	38.6	2.43	1.46-4.06	< 0.001
Patients with untreated anemia	13.8	16.8	1.21	0.63-2.32	0.559
Time with untreated anemia	73.9	11.3	0.15	0.1-0.23	< 0.001
Hemoglobin within goal	92.0	92.8	1.008	0.97-1.03	0.614
**Lipid abnormalities**					
Baseline hypercholesterolemia	61.4	59.4	0.92*	0.52-1.61	0.774
Hypercholesterolemia in follow-up	76.2	78.2	1.02	0.88-1.19	0.737
Patients with untreated Hypercholesterolemia	40.6	38.6	0.95	0.67-1.33	0.773
Time with untreated hypercholesterolemia	22.9	15.7	0.68	0.52-0.89	0.005
Total cholesterol within goal	66.4	69.9	1.05	0.97-1.13	0.188
Baseline elevated LDL cholesterol	82.0	76.2	0.7*	0.35-1.39	0.316
Elevated LDL cholesterol in follow-up	92.0	88.1	0.95	0.87-1.05	0.358
Patients with untreated elevated LDL	60.6	51.5	0.83	0.65-1.06	0.15
Time with untreated elevated LDL	35.6	31.0	0.87	0.73-1.03	0.117
LDL cholesterol within goal	40.3	45.7	1.13	0.98-1.3	0.07
Baseline hypertriglyceridemia	28.0	37.6	1.55*	0.85-2.8	0.147
Hypertriglyceridemia in follow-up	51.0	78.2	1.53	1.23-1.9	< 0.001
Triglyceridemia within goal	67.7	58.2	0.85	0.78-0.93	< 0.001
**Bone mineral disorder**					
Baseline hyperphosphatemia	5.5	4.9	0.89*	0.25-3.2	0.865
Hyperphosphatemia in follow-up	12.7	19.8	1.55	0.8-2.99	0.191
Patients w/ untreated hyperphosphatemia	7.4	18.8	2.52	1.11-5.73	0.026
Time with untreated hyperphosphatemia	30.0	56.1	1.86	1.01-3.44	0.044
Phosphataemia within goal	94.9	95.5	1.006	0.97-1.03	0.654
Baseline hyperparathyroidism	1.4	2.9	2.05*	0.18-23.2	0.559
Hyperparathyroidism in follow-up	5.7	4.3	0.76	0.17-3.27	0.713
Patients with untreated hyperparathyroidism	2.8	4.4	1.52	0.26-8.82	0.639
Time with untreated hyperparathyroidism	30.0	25.0	0.83	0.24-2.8	0.768
PTH within goal	95.3	95.7	1.003	0.95-1.05	0.873
Baseline hypovitaminosis D	76.1	59.2	0.45*	0.18-1.13	0.091
Hypovitaminosis D in follow-up	84.1	77.8	0.92	0.74-1.15	0.489
Patients with untreated hypovitamin D	53.4	66.6	1.24	0.89-1.73	0.188
Time with untreated hypovitamin D	28.3	33.3	1.17	0.82-1.68	0.368
25-OH Vitamin D within goal	39.2	49.4	1.25	0.96-1.64	0.09
**Other metabolic parameters**					
Baseline hyperuricemia	30.3	14.0	0.37*	0.18-0.76	0.006
Hyperuricemia in follow-up	40.4	39.0	0.96	0.68-1.35	0.839
Patients with untreated hyperuricemia	28.3	34.0	1.2	0.79-1.82	0.385
Time with untreated hyperuricemia	35.4	60.4	1.7	1.31-2.21	< 0.001
Uricemia within goal	83.9	87.2	1.03	0.98-1.09	0.146
Baseline metabolic acidosis	17.5	19.4	1.13*	0.46-2.78	0.783
Metabolic acidosis in follow-up	28.1	34.7	1.23	0.73-2.08	0.425
Patients with untreated metabolic acidosis	3.5	12.5	3.56	0.8-15.84	0.095
Time with untreated metabolic acidosis	2.6	8.5	3.3	0.74-14.7	0.116
Serum bicarbonate within goal	86.4	90.1	1.04	0.96-1.13	0.316

^1^Data are shown as percentages. Frequencies were compared using Chi-square or Fisher's test. ^2^NT-PDP: non-transplanted pre-dialysis patients; KTR: kidney transplant recipients; OR: odds ratio; RR: relative risk; CI: confidence interval; BP: blood pressure. LDL: low-density lipoprotein; HDL: high-density lipoprotein.

* OR (odds ratio).

Baseline median proteinuria (Table 1) and prevalence of significant proteinuria throughout follow-up were similar between groups (Table 3). A trend toward longer periods of untreated significant proteinuria was observed in KTR (17.2 *vs*. 7.5%, RR 2.27, CI 0.76-6.73, *p* = 0.137), although KTR were more often within goal (92.7 *vs*. 83.5%, RR 1.1, CI 1.05-1.16, *p* < 0.001) (Table 3).

At baseline, mean hemoglobin was significantly higher in NT-PDP (13.5±1.5 vs. 12.7±1.6 g/dL, *p* < 0.001) (Table 1). The prevalence of anemia was higher in KTR at baseline (22.8 *vs*. 6.9%, OR 3.95, CI 1.61-9.71, *p* < 0.001) and during follow-up (38.6 vs 15.8%, RR 2.43, CI 1.46-4.06, *p* < 0.001); however, the overall percentage of untreated patients was not statistically different between groups. Conversely, the length of time with untreated anemia was much lower for KTR than NT-PDP (11.3 *vs*. 73.9%, OR 0.15, CI 0.1-0.23, *p* < 0.001) (Table 3). Both cohorts, however, remained within desired hemoglobin goals for over 92% of the follow-ups, with no difference between them (Table 3).

Baseline HDL tended to be higher in NT-PDP (50.4±14.7 *vs*. 46.4±13.5 mg/dL, *p* = 0.056), but total cholesterol and LDL were similar (Table 1). The prevalence of hypercholesterolemia and elevated LDL was similar between groups, both at baseline and during follow-up (Table 3). NT-PDP endured longer periods without treatment of hypercholesterolemia (22.9 *vs*. 15.7%, OR 0.68, CI 0.52-0.89, *p* = 0.005), although both cohorts were within specified total cholesterol goals during follow-up (Table 3). LDL values and untreated elevated LDL were not different between groups (Table 3). Conversely, among KTR, hypertriglyceridemia tended to be more common at baseline, was more common throughout follow-up (78.2 *vs*. 51.0%, OR 1.53, CI 1.23-1.9, *p* < 0.001), and was less often within goal (58.2 *vs*. 67.7%, OR 0.85, CI 0.78-0.93, *p* < 0.001) (Table 3).

Baseline calcium levels were statistically, though not clinically, different between groups (9.4±0.7 in NT-PDP *vs*. 9.7±0.7 mg/dL in KTR, *p* = 0.033), and there were no differences in baseline phosphorus, PTH, and 25-OH-vitamin D between groups (Table 1). Prevalence of hyperphosphatemia was similar at baseline and, although time spent with untreated hyperphosphatemia was significantly higher in KTR (56.1 vs. 30.0%, RR 1.86, CI 1.01-3.44, *p* = 0.044), both cohorts spent approximately 95% of time within goal (Table 3).

Hyperparathyroidism requiring clinical treatment (> 450 pg/mL) was rather uncommon (5.7% in NT-PDP and 4.3% in KTR). No differences in treatment were observed, and both groups remained within PTH goals for over 95% of follow-up (Table 3). Vitamin D deficiency tended to be observed more frequently in NT-PDP at baseline (76.1 vs. 59.2%, OR 0.45, CI 0.18-1.13, *p* = 0.091), which was not observed during follow-up. Although the time with untreated deficiency was similar between groups, KTR were more often within goal (49.4 *vs*. 39.2%, RR 1.25, CI 0.96-1.64, *p* = 0.09) (Table 3).

Baseline uricemia was similar between groups. Although prevalence of hyperuricemia was higher in NT-PDP (30.3 *vs*. 14.0%, OR 0.37, CI 0.18-0.76, *p* = 0.006), this difference was not observed during follow-up. KTR had longer untreated hyperuricemia (60.4 *vs*. 35.4%, RR 1.7, CI 1.31-2.21, *p* < 0.001), and time within goal was similar between groups (Table 3). Prevalence of metabolic acidosis was similar between groups at baseline and during follow-up. While untreated metabolic acidosis tended to be observed more often in KTR, serum bicarbonate was similarly within range in both groups during follow-up (Table 3).

**Figure 2:**
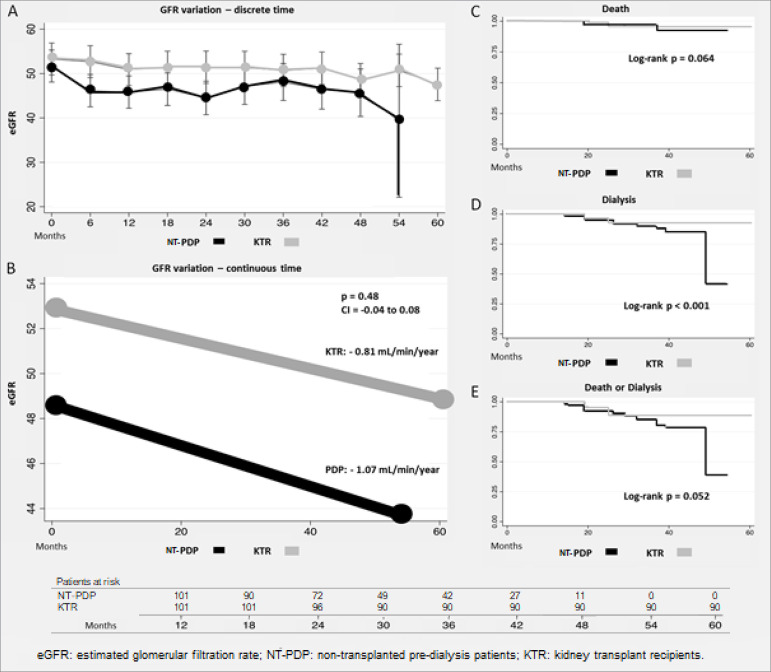
Glomerular filtration rate variation with discrete (A) and continuous time (B), and Kaplan-Meier curves for death (C), dialysis (D), and death or dialysis (E). eGFR: estimated glomerular filtration rate; NT-PDP: non-transplanted pre-dialysis patients; KTR: kidney transplant recipient.

Kaplan-Meier analysis revealed comparable mortality between the cohorts (3.9% in both cohorts, *p* = 0.064) (Figure 2). Infections were more common in KTR (50.4 *vs*. 6.9%, *p* < 0.001), cardiovascular events were uncommon in both (0.9% in KTR *vs*. 1.9% in NT-PDP, *p* = 0.56), as was cancer (5.9% in NT-PDP *vs*. 2.9% in KTR, *p* = 0.306). GFR decay was low and not different between groups (0.81 mL/min/year in KTR vs. 1.07 mL/min/year in NT-PPD, *p* = 0.48, CI 0.04-0.08) (Figure 2). NT-PDP progressed more often to dialysis (9.9% *vs*. 6.9%, *p* < 0.001), and the survival for a combined endpoint of death and dialysis tended to be worse among NT-PDP (13.9% *vs*. 10.9%, *p* = 0.052) (Figure 2). Patients from both cohorts who progressed to dialysis were younger, with lower baseline GFR, and more often had glomerulonephritis as the primary cause of CKD (Table 4).

**Table 4:** Demographic characteristics and progression to dialysis

	NT-PDP progressed to dialysis (N = 10)	NT-PDP not progressed to dialysis (N = 91)	KTR progressed to dialysis (N = 7)	KTR not progressed to dialysis (N = 94)
**Primary cause of CKD**				
Glomerulonephritis	60.0	7.7	57.1	39.3
Hypertension	20.0	46.1	0.0	22.3
Diabetes	10.0	3.3	14.3	1.1
Polycystic kidney disease	0.0	13.2	0.0	5.3
Other	10.0	6.6	14.3	5.3
Undetermined	0.0	23.1	14.3	26.6
**Baseline renal function**				
Creatinine (mg/dL)	2.58 ± 0.89	1.51 ± 0.47	2.24 ± 1.00	1.54 ± 0.45
eGFR (mL/min/1.73m^2^)	33.4 ± 12.4	53.7 ± 19.9	37.23 ± 13.0	54.4 ± 16.6
**Demographics**				
Female gender (%)	30.0	38.5	57.1	29.8
Age (years)	36.6 ± 11.0	51.8 ± 12.9	36.3 ± 11.8	43.9 ± 12.5
**Donor type (%)**				
Living related	-	-	85.7	85.1
Living unrelated	-	-	14.3	11.7
Deceased	-	-	0.0	3.2

^1^Data are shown as percentages or means ± standard deviation. ^2^NT-PDP: non-transplanted pre-dialysis patients. KTR: kidney transplant recipients. eGFR: estimated glomerular filtration rate, according to the CKD-EPI formula. CKD: chronic kidney disease.

## Discussion

Multidisciplinary teamwork provides better long-term results for patients with chronic conditions such as CKD.^18^ KTR are a particular subset of patients in which CKD-related complications and risk factors for disease progression concur with major immunological concerns and the use of immunosuppression drugs.^10^ The importance of multidisciplinary approach in KTR has been suggested by studies comparing multidisciplinary and non-multidisciplinary post-transplant clinics.^14^ In our retrospective study, we compared KTR and NT-PDP groups after PSM, both under multidisciplinary follow-up. The cohorts had similar eGFR, CKD category distribution, and prevalence of diabetes and cardiovascular comorbidities, but KTR were younger and had longer follow-up. Time within most therapeutic goals was similar between groups, with the exception of DBP and triglyceridemia, controlled for longer in NT-PDP, and proteinuria, controlled for longer in KTR. Patient survival and GFR decay were similar between groups, although NT-PDP progressed earlier to dialysis.

Anemia was more common and treated more often in KTR, partly because most anemic KTR were already using erythropoietin at the study onset, whereas most NT-PDP were incident patients. The similar absolute percentage of patients from each cohort with untreated anemia at any point in time (13.8% *vs*. 16.8%, *p* = 0.559) reinforces that observation. Akbari et al., in a transversal study, described worse results, with 59% of KTR without multidisciplinary care, and 21% of NT-PDP with multidisciplinary care, to be with untreated anemia.^13^

The finding of proteinuria in KTR being more often controlled should be considered with caution, since proteinuria in NT-PDP usually has a different meaning than in KTR. Especially after 1 year post-transplant, proteinuria could represent a number of concurrent conditions implied in tubulo-interstitial derangement of the graft, in the context of multifactorial chronic allograft nephropathy, such as alloimmune response, recurrent or *de novo* glomerulonephritis, or adverse effects of immunosuppressive medications, notably mammalian target of rapamycin inhibitors (m-TORi).^19^^,^ 20

Interestingly, we observed a downslope of eGFR in the first year of follow-up in NT-PDP, followed by a less steep curve, probably reflecting the introduction and dose adjustments of antihypertensive medications, particularly angiotensin-converting enzyme inhibitors (ACEi) or angiotensin receptor blockers (ARB), in incident patients entering the NT-PDP cohort. This observation is paralleled by mean SBP and DBP being higher in PDP at baseline, which was not persistent throughout follow-up. Overall, both cohorts had SBP and DBP controlled for over 75% of the observation period. Due to the variable nature of available studies, straight comparisons cannot be drawn between their results and ours. Carpenter et al. described controlled BP (< 130/80 mmHg) in 56% of KTR, whereas Bissonnette et al. found 65% of SBP and 88% of DBP to be controlled in KTR with GFR < 30 mL/min/m^2^ under multidisciplinary care. Akbari et al. reported 40% of KTR in category 5 of CKD had controlled BP without multidisciplinary care.^13^^,^ 14 ^,^ 21

The higher prevalence and poorer control of hypertriglyceridemia in KTR were probably related to side effects of immunosuppressive drugs, such as prednisone, calcineurin inhibitors, and mammalian-target of rapamycin inhibitors (m-TORi).^22^ Although untreated hypercholesterolemia was less often observed in KTR, concerns regarding avoidance of polypharmacy, potentially harmful drug interactions, and adverse drug effects may have prevented the use of fibrates in KTR. Similar findings were reported by Akbari et al., who described hypertriglyceridemia in 67% of KTR without multidisciplinary treatment and in 50% of NT-PDP under multidisciplinary care. In the present study, we observed hypertriglyceridemia in 67.7% of KTR and 58.2% of NT-PDP, despite multidisciplinary follow-up in both cohorts.^13^

Polypharmacy avoidance could also partly explain why KTR had untreated hyperphosphatemia and hyperuricemia for longer periods. However, no differences were observed in the percentage of clinical visits wherein both phosphate and uric acid were within goals for both cohorts. Again, as a comparison, Akbari et al. described untreated hyperphosphatemia in 71.4% of category 5 KTR in a non-multidisciplinary setting, and in 13.3% of category 5 NT-PDP under multidisciplinary care.^13^ Bissonnette et al., later described the use of phosphate chelators in 73% KTR under multidisciplinary treatment, as opposed to 25% KTR in non-multidisciplinary setting, despite the ease of attaining clinical targets for hyperphosphatemia in both cohorts (90% and 85%, respectively, without statistical difference).^14^

Patient survival was similar between groups. The observed GFR decline was very slow and similar in both cohorts, although NT-PDP progressed to dialysis earlier.^3^^,^ 23 Considering the nature of the KTR we studied, who mostly received living, related-donor grafts, and whose characteristics led to the NT-PDP cohort selected through PSM, the results we described must be carefully compared to those from other authors.^7^^,^ 13 Still, we demonstrated that throughout follow-up, the percentage of time within most therapeutic goals was similar between groups, indicating a positive result based on the hypothesis that multidisciplinary care could provide high quality treatment for CKD in KTR, similarly to NT-PDP, as previously suggested.^13^^,^ 18

Some important limitations of this study include its single-center, retrospective non-randomized study design, and its relatively small sample of KTR compared to mostly incident NT-PDP. This may account for the protection against hard endpoints in the KTR group and also limits the inference about CKD progression. To correct the demographic disparities, we employed "nearest neighbor" PSM, obtaining the best possible sample of NT-PDP from a larger cohort to match the KTR.^16^ Despite not being able to fully match the cohorts for age, BMI, and length of follow-up, we were able to equalize both cohorts in terms of GFR and CKD stage, the prevalence of diabetes, and cardiovascular comorbidities. Considering there is no strong evidence supporting the beneficial effect of CKD management in KTR, we chose to adopt, as KDIGO 2012 suggests, the current CKD treatment recommendations for the KTR and NT-PDP.^5^ Few well-designed studies have described the beneficial impact of multidisciplinary compared to non-multidisciplinary care on KTR, and only one cross-sectional study has demonstrated comparable quality of multidisciplinary treatment of CKD-related complications between NT-PDP and KTR.^13^^,^ 14 ^,^ 24 The present study is the first to compare the quality of treatment of CKD-related complications throughout a specified follow-up period, between KTR and NT-PDP cohorts, when both were under multidisciplinary care. Besides, this is one of the few studies about CKD treatment in a Brazilian KTR population.

In conclusion, the percentage of time spent within most therapeutic goals was similar between the cohorts. Despite being based on a small sample, we found comparable patient survival, and GFR decline, although NT-PDP more often progressed to dialysis. The observed results suggest that multidisciplinary clinics could contribute for good quality follow-up of KTR.
